# Molecular pathways linking traumatic brain injury to cardiovascular dysfunction: therapeutic potential of physical exercise

**DOI:** 10.1007/s11011-026-01880-2

**Published:** 2026-06-09

**Authors:** Leandro Machado Severo Feiteiro, Eduardo Felipe Alchieri, Douglas Buchmann Godinho, Giovana Dal Magro, Julia Torbes, Derick Minuzzi Cardozo, João Victor de Mattos Mautone Pedroso, Ana Flávia Furian, Mauro Schneider Oliveira, Michele Rechia Fighera, Luiz Fernando Freire Royes

**Affiliations:** 1 Centro de Ciências Naturais e Exatas, Programa de Pós Graduação em Ciências Biológicas: Bioquímica Toxicológica, Universidade Federal de Santa Maria, Santa Maria - RS, Brasil; 2 Centro de Ciências da Saúde, curso de Medicina, Universidade Federal de Santa Maria, Santa Maria - RS, Brasil; 3 Centro de Ciências da Saúde, Departamento de Neuropsiquiatria, Universidade Federal de Santa Maria, Santa Maria - RS, Brasil; 4 Centro de Educação Física e Desportos, Laboratório de Bioquímica do Exercício (BIOEX), Universidade Federal de Santa Maria, Santa Maria - RS, Brasil; 5 Centro de Educação Física e Desportos, curso de Educação Física Bacharelado, Universidade Federal de Santa Maria, Santa Maria - RS, Brasil; 6 Centro de Ciências Rurais, Departamento de Tecnologia e Ciência dos Alimentos, Universidade Federal de Santa Maria, Santa Maria - RS, Brasil; 7 Centro de Ciências da Saúde, Departamento de Fisiologia e Farmacologia, Universidade Federal de Santa Maria, Santa Maria - RS, Brasil; 8 Centro de Educação Física e Desportos, Departamento de Métodos e Técnicas Desportivas, Universidade Federal de Santa Maria, Santa Maria, 97105-900 RS Brasil

**Keywords:** Traumatic brain injury, Autonomic nervous system, Cardiovascular dysfunction, Physical exercise, Neurocardiovascular axis, Rehabilitation

## Abstract

Traumatic brain injury (TBI) affects millions globally and increasingly associates with cardiovascular disease risk through mechanisms involving autonomic nervous system dysfunction, neuroinflammation, oxidative stress, and hypothalamic-pituitary axis disruption. However, the precise molecular pathways remain incompletely understood. This review synthesizes current evidence on TBI-induced cardiovascular dysfunction and explores physical exercise as a therapeutic intervention. We conducted a comprehensive literature narrative review of experimental and clinical studies investigating cardiovascular complications following TBI and exercise-based interventions. TBI triggers a cascade of molecular events leading to autonomic imbalance, chronic inflammation, and oxidative damage that collectively increase cardiovascular risk. Physical exercise emerges as a promising intervention through its capacity to modulate inflammatory processes, restore autonomic balance, and enhance synaptic plasticity. However, optimal timing, intensity, and duration of exercise protocols remain undefined. Understanding heart-brain communication pathways post-TBI is crucial for developing targeted interventions. Physical exercise shows therapeutic potential, but standardized protocols are needed to optimize cardiovascular outcomes in TBI patients.

**Graphical Abstract:**

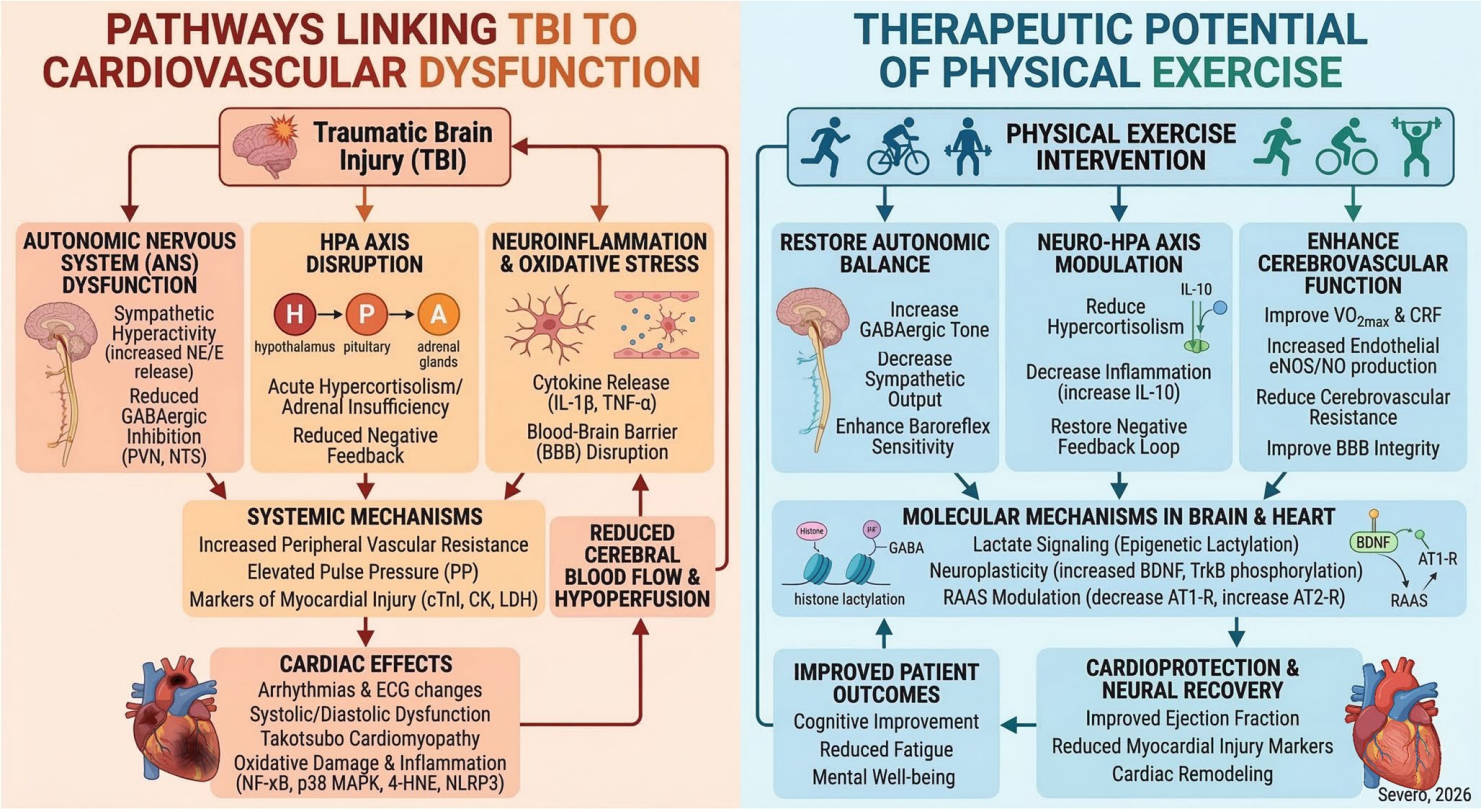

## Introduction

Traumatic brain injury (TBI) is an alteration in brain function caused by an external mechanical force (Blennow et al. 2016). This condition affects an estimated 64 to 74 million people annually, making it a major global public health burden (Dewan et al. 2019). TBI involves complex pathophysiological processes, which are typically categorized into two phases: primary and secondary injury. The primary injury occurs at the moment of impact, when an external mechanical force affects the brain, directly impacting the skull, face, or any other part of the body that transmits an impulsive force to the head (McCrory et al. 2013; Meyfroidt et al. 2022). Secondary injury is characterized by a cascade of metabolic and biochemical alterations that can persist for hours, days, months, or even years after the initial trauma (Blennow et al. 2016). Although neurological outcomes have received primary research attention, TBI also triggers systemic physiological alterations affecting peripheral organs, including the gut, liver, pancreas, spleen, and cardiovascular system (Buchmann Godinho et al. 2021).

After TBI, systemic dysfunction characterized by autonomic nervous system (ANS) imbalance leads to heart rate instability, reduced heart rate variability (HRV), and increased risk of cardiac arrhythmias (Purkayastha et al. 2019). The ANS plays a critical role in the stress response and neurocardiovascular integration; consequently, TBI-induced catecholamine release from hypothalamic and brainstem regions results in sympathetic hyperactivity (Zhao et al. 2019). This pathophysiological mechanism is supported by randomized clinical trials demonstrating that beta-blocker administration within 24 h of severe TBI significantly reduces mortality rates and improves functional outcomes six months post-injury (Khalili et al. 2020). These protective effects may result from reduced cerebral metabolism, which decreases oxygen demand and mitigates ischemia secondary to the initial trauma (Chen et al. 2017). Additionally, preclinical studies have demonstrated that TBI-induced cardiovascular reactivity and behavioral deficits can be prevented by administering alpha-methyltyrosine (αMT), a catecholamine synthesis inhibitor (Woodman et al. 2023). Furthermore, the acceleration of arteriosclerosis progression through beta-adrenergic activation in murine TBI models reinforces the involvement of the sympathetic system in this neurological injury’s pathophysiology (Wang et al. 2023).

Cardiac function is regulated by multiple physiological mechanisms, including autonomic nervous system control, endocrine signaling, and hemodynamic factors (Keane et al. 2023a; Plott et al. 2024; Prince et al. 2024). Following TBI, synaptic reorganization occurs due to excessive glutamate release and reduced γ-aminobutyric acid (GABA) levels, disrupting the balance between excitatory and inhibitory signaling (Dobrachinski et al. 2017a; Gerbatin et al. 2017). This excitatory-inhibitory imbalance promotes sympathetic hyperactivity in brainstem regions that regulate cardiac function. Supporting this mechanism, studies have documented post-TBI cardiac abnormalities, including arrhythmias and ECG changes, along with elevated blood levels of cardiac troponin (cTnI) and creatine kinase (CK), markers of myocardial injury (Coppalini et al. 2024). In parallel, TBI triggers elevated inflammatory markers, oxidative stress, and altered calcium signaling in cardiac tissue (Keane et al. 2023a; Peng et al. 2023). These cardiac changes promote increased peripheral vascular resistance and reduced cerebral blood flow, which may impede cognitive and psychiatric rehabilitation processes (Tomoto et al. 2022). However, these mechanisms remain poorly characterized, and a comprehensive understanding of the heart-brain axis in TBI pathophysiology may inform targeted therapeutic interventions for post-traumatic recovery. Physical exercise represents a promising therapeutic approach, given its established capacity to induce beneficial long-term physiological adaptations (Snowden et al. 2023).

In this regard, planned, structured, and repetitive physical exercise promotes beneficial cardiovascular, neurological, and metabolic adaptations (Bull et al. 2020a). These changes are associated with improvements in myocardial oxygen demand, autonomic tone, and inflammatory markers (Todhunter-Brown et al. 2025). In the context of TBI, cognitive and physical rest until symptom resolution has traditionally been prescribed, a practice currently being questioned (Snowden et al. 2023). Evidence suggests that early and gradual return to physical activity may accelerate neurological recovery (Weldon et al. 2024a). Along these lines, studies demonstrate that aerobic exercise protocols improve cognitive function and oxygen uptake kinetics in post-TBI patients (Chin et al. 2015a, 2019a). However, the mechanisms by which different exercise protocols modulate the pathological outcomes of TBI remain partially elucidated. From this perspective, this narrative review aims to examine the mechanisms by which physical exercise influences post-TBI neurological outcomes, addressing: (1) pathophysiological aspects of TBI, (2) the relationship between traumatic brain injury and cardiac disorders, (3) physical exercise in the treatment of heart diseases, and (4) physical exercise as a non-pharmacological approach to treat TBI-induced heart diseases. This integrative analysis seeks to provide theoretical foundations for improving rehabilitation protocols in patients with TBI.

## Method

### Inclusion criteria for clinical studies

Physical Exercise interventions must include a plan, structured, repeated with maintenance of physical fitness or health. In addition, studies were limited to randomized control trials (RCTs) or with pre and post-intervention groups with predictors of cardiorespiratory fitness (direct and indirect). The emphasis of the studies was on trauma-induced brain injuries with severity (mild, moderate, and severe), both sexes, in the adolescent and adult phases. The search strategy was conducted in electronic databases, including PubMed, Scopus, and Google Scholar, utilizing a combination of search terms (see Table 1). Due to the low number of clinical studies meeting the inclusion criteria, the searches were limited to the period from 2010 to 2025.

**Table 1:** keywords used for the literature search

Set	Search Terms
#1	(((exercise OR physical exercise) AND (Traumatic brain injury OR TBI) AND (Cardiorespiratory fitness OR cardiovascular disease)))
#2	((exercise OR physical exercise) AND (Traumatic brain injury OR TBI))
#3	((Traumatic brain injury OR TBI) AND (Cardiorespiratory fitness OR cardiovascular disease)).
#4	((exercise OR physical exercise) AND (Cardiorespiratory fitness OR cardiovascular disease)).

### The pathophysiology of TBI

Traumatic brain injury (TBI) is characterized by altered brain function resulting from external mechanical forces. TBI is classified as either penetrating, involving skull perforation and direct brain parenchyma damage, or non-penetrating, where the skull remains intact. In non-penetrating cases, damage results from acceleration-deceleration forces that cause microlesions in brain tissue (Blennow et al. 2016). Along these lines, injury severity is commonly assessed using the Glasgow Coma Scale (GCS): mild (13–15), moderate (9–12), or severe (3–8) (Teasdale and Jennett, 1974).

Epidemiological data indicate that TBI affects approximately 74 million people annually, representing a major global public health concern (Dewan et al. 2019). Beyond acute care, these injuries result in long-term physical, behavioral, and emotional impairments that place substantial burden on healthcare systems. In the United States alone, an estimated 5.3 million Americans live with TBI-related disabilities, often accompanied by comorbid conditions including alcoholism, epilepsy, depression, and neurodegenerative disorders such as Alzheimer’s disease (Dewan et al. 2019). Regarding etiology, falls represent the leading cause (35%), followed by motor vehicle collisions (17%), sports-related incidents (16.5%), and assaults (10%), with the remaining 21% attributed to other or unknown factors (Faul and Coronado 2015).

The pathophysiology of TBI is typically categorized into two phases: primary and secondary injury. Primary injury refers to the immediate mechanical damage occurring at the moment of impact, resulting in diffuse tissue deformation or focal lesions from penetrating objects (Blennow et al. 2016). In contrast, secondary injury involves a delayed cascade of pathological events, including excitotoxicity, mitochondrial dysfunction, neuroinflammation, and oxidative stress (Buchmann Godinho et al. 2021; Gerbatin et al. 2017; Severo et al. 2020).

The initial cellular response to TBI involves massive depolarization of neurons and glial cells triggered by mechanical energy transfer to brain tissue (Barkhoudarian et al. 2016). This depolarization causes excessive potassium (K+) efflux and subsequent glutamate release, hyperactivating postsynaptic N-methyl-D-aspartate (NMDA) and α-amino-3-hydroxy-5-methyl-4-isoxazolepropionate (AMPA) receptors (Dobrachinski et al. 2017b; McKee and Daneshvar, 2015). This excessive glutamate release overwhelms the mitochondrial calcium ion (Ca²⁺) buffering capacity, leading to mitochondrial permeability transition pore (mPTP) opening and reduced adenosine triphosphate (ATP) synthesis (Courtes et al. 2020; Gerbatin et al. 2017). Additionally, mitochondrial dysfunction increases reactive oxygen species (ROS) production, contributing to cell death through both direct apoptotic mechanisms and indirect pathways (Lazzarino et al. 2019; Sullivan et al. 2005).

Concurrently, TBI-induced cellular hypoxia necessitates a metabolic shift toward anaerobic glycolysis, resulting in lactate accumulation and subsequent perturbation of cerebral energy homeostasis (Severo et al. 2020). Another critical component of secondary injury is the disruption of the blood-brain barrier (BBB), which leads to vasogenic edema and triggers a neuroinflammatory response. This response is characterized by the activation of microglia, which adopt a pro-inflammatory phenotype and release a cascade of cytokines, including interleukin 1β (IL-1β), IL-6, IL-17, tumor necrosis factor-α (TNF-α), and interferon-γ (IFN-γ) (Rajendram et al., 2022). Collectively, this complex neurochemical cascade disrupts the function of critical regulatory systems, including the autonomic nervous system (ANS), the hypothalamic-pituitary-adrenal (HPA) axis, and the renin-angiotensin system (RAS) (Mahajan et al. 2023; Villapol et al. 2023).

The secondary injury cascade following TBI does not occur as isolated parallel pathways, but rather as an integrated, sequential pathological continuum (Blennow et al. 2016). The initial mechanical impact triggers massive cellular depolarization and glutamate excitotoxicity, which overwhelms mitochondrial calcium buffering capacity (Barkhoudarian et al. 2016; Courtes et al. 2020). This immediate mitochondrial dysfunction and subsequent ATP depletion generate a surge in reactive oxygen species (ROS) (Lazzarino et al. 2019; Sullivan et al. 2005). Acting in synergy, this oxidative stress and cellular damage promote blood-brain barrier (BBB) disruption and microglial activation, establishing a robust neuroinflammatory microenvironment characterized by the release of IL-1β and TNF-α (Rajendram et al., 2022; Kim et al. 2024).

The combined effects of oxidative damage and sustained neuroinflammation impair the structural and functional integrity of neural networks, leading to a reduction in GABAergic inhibitory tone, particularly within key autonomic centers such as the paraventricular nucleus (PVN) of the hypothalamus (Feng et al. 2024; Woodman et al. 2023). The loss of this central inhibitory control serves as the definitive mechanistic bridge to systemic dysfunction, directly unleashing sympathetic hyperactivity in the rostral ventrolateral medulla (RVLM) and disrupting the HPA axis (Faden et al. 2021; Kim et al. 2015). In this scenario, a continuous cascade beginning with mitochondrial failure, progressing through neuroinflammation, and culminating in autonomic dysregulation leads to excessive catecholamine release, which ultimately drives the secondary myocardial injury observed after TBI (Li 2022; Peng et al. 2023).

### The relationship between traumatic brain injury and cardiac disorders

Epidemiological studies indicate that up to 83.2% of patients with TBI sustain concurrent extracranial injuries (Greil et al. 2024). Among these patients, approximately 10% are at risk of cardiac arrest, particularly those with lower Glasgow Coma Scale (GCS) scores. This risk is underscored by a strong correlation between the severity of neurological injury and serum levels of myocardial injury biomarkers, such as troponin T and C-reactive protein. This link is further supported by findings that propranolol treatment in TBI patients reduces both epinephrine concentrations and inflammatory biomarkers (IL-6, IL-18, and IL-1β), suggesting that post-TBI cardiac dysfunction is driven by epinephrine-mediated inflammatory responses (El-Menyar et al. 2024). Indeed, post-TBI catecholamine system dysfunction has been clinically linked to a range of cardiac manifestations, including systolic dysregulation, QT interval prolongation, supraventricular arrhythmias, myocardial stunning, and Takotsubo cardiomyopathy (TC) (Khacho et al. 2019; Khalili et al. 2020). Takotsubo cardiomyopathy, an acute heart failure syndrome characterized by electrocardiographic changes and elevated myocardial biomarkers in the absence of obstructive coronary artery disease, can be directly induced by TBI (Cheah et al. 2017).

This complex brain-heart interaction highlights the therapeutic potential of β-blockers, which have shown promise in mitigating adverse cardiac events, reducing circulating catecholamine levels, and correcting cerebral metabolic dysfunction. Furthermore, this drug class may alleviate metabolic acidosis and enhance cerebral perfusion (Gruhl et al. 2022). A growing body of evidence indicates that TBI-induced autonomic dysfunction is a major contributor to multiorgan failure, thereby exacerbating the primary pathophysiology (Royes and Gomez-Pinilla 2019). In this scenario, a significant proportion of TBI patients have been reported to experience electrolyte imbalances, which have been associated with pituitary gland dysfunction. Considering that brain injuries primarily affect the hypothalamic-pituitary-adrenal (HPA) axis and catecholamine systems, it is plausible to propose that changes in cardiac function elicited by HPA axis dysfunction exacerbate responses to secondary injuries and hinder neurological recovery after TBI. This assumption is supported by experimental studies in TBI models that observed upregulation of immediate early genes (IEGs) along the HPA axis (e.g., pituitary, adrenal glands) and other peripheral organs (e.g., heart), resulting in changes in body weight, memory function, locomotion and stress response (Reiners et al. 2023).

### TBI-induced autonomic nervous system dysfunction

The autonomic nervous system (ANS) orchestrates involuntary physiological processes through its two main branches: the sympathetic (SNS) and parasympathetic (PNS) nervous systems. The ANS utilizes afferent (sensory) and efferent (motor) fibers to integrate signals from the body and control organ function (Gordan et al. 2015).

**Fig. 1:**
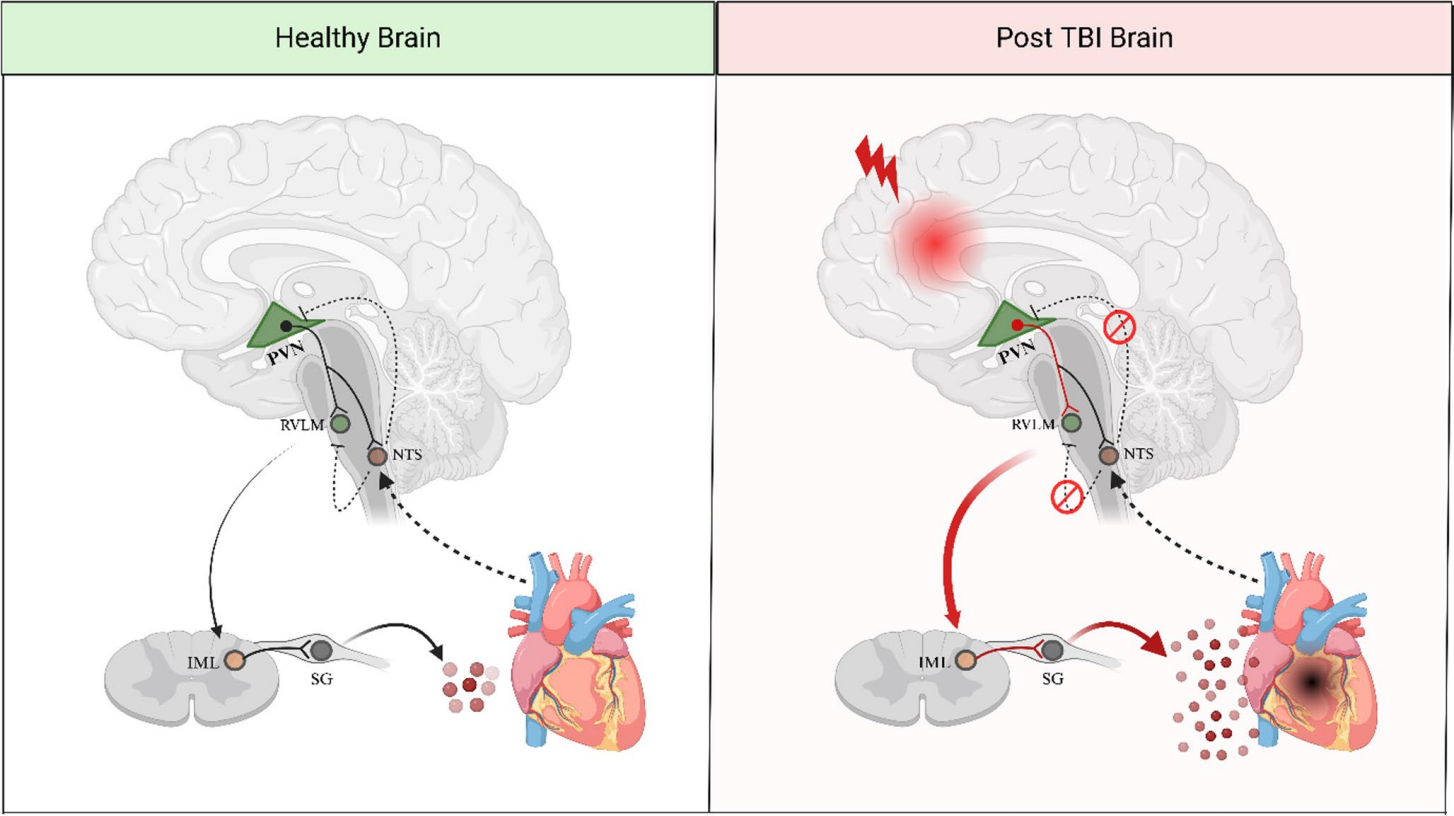
Schematic Illustration of autonomic nervous system dysfunction induced by traumatic brain injury (TBI)

The SNS mediates the “fight or flight” response via the release of catecholamines, primarily norepinephrine (NE) and epinephrine. These neurotransmitters increase heart rate and blood pressure, promote glycogenolysis, and inhibit gastrointestinal motility (Waxenbaum et al. 2025). Central control of sympathetic outflow to the heart is hierarchical (Fig. 1). The paraventricular nucleus (PVN) of the hypothalamus, a key integration center, projects to autonomic nuclei in the brainstem, including the rostral ventrolateral medulla (RVLM) and the nucleus of the solitary tract (NTS) (Hubens et al. 2013). From the brainstem, these pathways descend to the intermediolateral (IML) column of the spinal cord, where they synapse onto preganglionic neurons that, in turn, activate postganglionic neurons innervating the heart (Li 2022). Norepinephrine released from these terminals acts on β-adrenergic receptors to modulate cardiac electrophysiology. Consequently, excessive sympathetic activation is a well-established contributor to cardiac pathologies, including hypertension, myocardial injury, Takotsubo syndrome, and ischemia (Liu et al. 2022a).

In the healthy brain, regarding cardiac functions, the SNS operates under a hierarchical regulatory system, in which the paraventricular nucleus (PVN) of the hypothalamus serves as a key center for autonomic regulation. The PVN projects efferent sympathetic circuits through the rostroventrolateral medulla (RVLM) and the nucleus of the solitary tract (NTS) in the brainstem. These neurocircuits subsequently innervate the intermediolateral column (IML) of the spinal cord in the thoracic region, which, in turn, modulates pre- and post-ganglionic sympathetic neurons. Finally, norepinephrine is released at nerve terminals, influencing cardiac electrophysiology through beta-adrenergic receptors. The balance between the SNS and PNS is integrated by the brain through baroreceptors located in the carotid sinus and aortic arch. The electrical signal travels along vagal afferent pathways, reaching the (NTS). Under healthy physiological conditions, the NTS projects inhibitory GABAergic interneurons to the RVLM, reducing the activity of sympathoexcitatory neurons and lowering mean arterial pressure (MAP). It is suggested that post-TBI dysfunctions are associated with a decrease in GABAergic circuits within the paraventricular nucleus (PVN), as well of the NTS interneurons that project inhibitory signals to the RVLM and hypothalamus. This reduction in inhibitory circuitry favors sympathetic hyperactivity following the injury. Created in BioRender. Rechia Fighera, M. (2025) https://BioRender.com/81k4ghc.

Conversely, the (PNS) counteracts sympathetic activity through acetylcholine (ACh) release. The PNS mediates bradycardia via hypothalamic modulation of vagal efferent pathways (Gordan et al. 2015). Within cardiac myocytes, this neurocircuitry operates through muscarinic type 2 (M2) receptors, which activate the βγ subunit of Gi proteins to modulate K+ channel conductance, ultimately attenuating sinoatrial node action potential frequency (Olshansky et al. 2008). Sympathetic-parasympathetic balance is maintained through central integration of baroreceptor signals from the carotid sinus and aortic arch, complemented by mechanoreceptor input from vascular wall distension. These afferent signals traverse vagal pathways to reach the (NTS). Under physiological conditions, the NTS activates inhibitory GABAergic interneurons that project to both the caudal ventrolateral medulla (CVLM) and rostral ventrolateral medulla (RVLM), thereby suppressing sympathoexcitatory neuronal activity and reducing mean arterial pressure (MAP) (Wehrwein and Joyner 2013).

Although the mechanisms underlying ANS dysfunction following TBI remain incompletely elucidated, clinical evidence demonstrates that sympathetic hyperactivity and parasympathetic hypoactivity occur across all TBI severities (Hilz et al. 2017). Recent experimental findings from our research group revealed that moderate TBI induced by the (FPI) model produced electrocardiographic alterations characterized by shortened R-wave peak-to-R-wave peak (RR) intervals compared to mild injury and control groups. Furthermore, animals with moderate TBI exhibited a significant increase in the high-frequency (HF)/low-frequency (LF) ratio relative to mild TBI, suggesting that sympathetic-vagal imbalance correlates proportionally with injury severity (Do Nascimento et al. 2023). In the controlled cortical impact (CCI) model, electrophysiological recordings similarly demonstrated that TBI-induced cardiac autonomic dysfunction results, at least partially, from increased stellate ganglion (SG) neuronal excitability and diminished intracardiac ganglion (ICG) neuronal activity. This phenotype correlates with decreased KCNQ2 mRNA transcription in SG neurons, which normally regulate M-type potassium (K+) currents and prevent sympathetic neuronal hyperexcitability (Oh et al. 2021). Consequently, post-TBI sympathetic-parasympathetic imbalance in cardiac regulation compromises cerebral blood flow (CBF) and induces tachycardia.

These cardiovascular perturbations contribute to diminished quality of life and pose substantial challenges for post-TBI rehabilitation (Coppalini et al. 2024). Cardiac remodeling may impair cerebral perfusion, thereby disrupting the bidirectional heart–brain dynamics. This assumption is supported by recent findings showing that coronary artery disease and hypertension are associated with cortical remodeling in regions such as the superior frontal, and lateral occipital cortex (Liao et al. 2024). These regions are crucial for cognitive control and emotional regulation, mediated by cortico-limbic circuits (Liao et al. 2024; Liu et al. 2022b; Tahsili-Fahadan and Geocadin 2017). In addition, chronic cerebral hypoperfusion and endothelial dysfunction secondary to heart disease can trigger neuronal injury and deleterious microvascular reorganization (Siedlinski et al. 2023). It is important to note that afferent pathways from the heart convey not only chemical but also mechanical and sensory signals related to arterial pressure via chemo‑ and baroreceptor activation. These signals reach the thalamus, which integrates peripheral physiological information and projects it to the insular cortex, a crucial region for hemodynamic regulation (Hu et al. 2023). In a preclinical study by De Montgolfier et al. (2019) demonstrated that amyloid-β (Aβ) deposition, triggered by elevated pulse pressure (PP) and vascular mechanical stress, increases p21 cell cycle protein expression and activates apoptotic pathways via caspase-3 cleavage, leading to cognitive decline (De Montgolfier et al. 2019). In an experimental model of severe TBI, increased expression of inflammasomes NLR family pyrin domain containing 1 and 3 (NLRP1, NLRP3) and IL‑1β was observed in both the cerebral cortex and the left ventricular myocardium three days after injury (Keane et al. 2023b). Although the mechanisms by which post-traumatic cardiac changes influence brain structures via afferent pathways remain incompletely understood, we propose that an acute reduction in inhibitory circuits and sympathetic hyperactivity following neuronal injury contribute to increased pulse pressure (PP). Sustained elevation of PP may exacerbate brain injury by promoting micro-hemorrhages and activating neuronal apoptosis in regions critical for emotional and cognitive behavior.

### The hypothalamic-pituitary-adrenal axis and the stress response

The hypothalamic-pituitary-adrenal (HPA) axis is the body’s primary neuroendocrine system for managing responses to stress. Activation of this axis culminates in the release of glucocorticoids from the adrenal glands. The cascade begins when neural inputs to the paraventricular nucleus (PVN) of the hypothalamus trigger the secretion of corticotropin-releasing hormone (CRH). CRH then travels to the anterior pituitary gland, where it stimulates the release of adrenocorticotropic hormone (ACTH) into the bloodstream. Upon reaching the adrenal glands, ACTH promotes the synthesis and release of glucocorticoids, primarily cortisol in humans (Leistner and Menke 2020). Under normal physiological conditions, this system is tightly regulated by a negative feedback loop. Circulating cortisol acts on both the hypothalamus and the pituitary gland to inhibit the secretion of CRH and ACTH, respectively. This mechanism ensures that the stress response is terminated and homeostasis is restored (DeMorrow 2018).

TBI-induced tissue and axonal damage can disrupt the dynamic regulation of the HPA axis. For instance, studies indicate that 27.5% to 34% of individuals with TBI experience pituitary dysfunction, while 25% to 75% develop adrenal insufficiency following severe TBI (Feng et al. 2021; Schneider et al. 2007). Recently, clinical data showed that acute hypercortisolism observed in the serum and cerebrospinal fluid (CSF) of patients who have suffered severe TBI is associated with cognitive impairment six months after the injury (Barton et al. 2021). It is important to note that adrenal insufficiency may be correlated with injury severity. This assumption is based on a clinical study that did not observe adrenal dysfunction in patients with mild TBI. However, 22.85% and 44.82% of patients who suffered moderate and severe TBI, respectively, presented with adrenal insufficiency (Li et al. 2024). Preclinical studies have shown that corticosterone levels in rats, the rodent equivalent of cortisol in humans, significantly decreased 56 days post-injury (Rowe et al. 2016).

While the precise role of the HPA axis in post-TBI cardiac complications is still being elucidated, insights can be drawn from its well-established involvement in conditions like depression, anxiety, and cerebral ischemia (Feng et al. 2024). In these conditions, chronically elevated cortisol levels in limbic regions, particularly the hippocampus, lead to glucocorticoid receptor (GR) desensitization. This desensitization impairs the negative feedback loop of the HPA axis, resulting in sustained hyperactivity. Furthermore, HPA axis hyperactivity reduces the synthesis of key neuroplasticity proteins like brain-derived neurotrophic factor (BDNF) (Anacker et al. 2011). This sustained hypercortisolism can also promote neuroinflammation by increasing pro-inflammatory cytokines (e.g., TNF-α, IL-1β) and directly activate the SNS via glutamatergic signaling in the PVN (Chen et al. 2017). This sympathetic overactivation may be compounded by the loss of inhibitory control within the PVN. Given that GABAergic synapses are highly prevalent in this nucleus, HPA-driven disruption of this inhibitory tone could impair baroreflex signaling between the NTS and the heart, compromising parasympathetic control of blood pressure (Feng et al. 2024). Beyond the CNS, HPA axis dysregulation has systemic consequences, including increased blood-brain barrier (BBB) permeability and the promotion of intestinal dysbiosis. This “leaky gut” phenomenon can facilitate the translocation of bacteria and endotoxins into the systemic circulation, which in turn can directly impact cardiac tissue through heightened inflammation and oxidative stress (Buchmann Godinho et al. 2021). Collectively, these interconnected pathways, spanning from receptor desensitization to systemic inflammation, underscore the critical need for therapeutic strategies that target the restoration of CNS-peripheral communication after TBI. This need is particularly urgent given that many TBI survivors exhibit elevated biomarkers of cardiac damage.

### Preclinical evidence of TBI-induced cardiac tissue alterations

From a metabolic perspective, alterations in critical brain regions such as the hypothalamus and brainstem following TBI have been shown to drive both sympathetic hyperactivation and HPA axis dysfunction (De Castro et al. 2017; Faden et al. 2021). As summarized in Table 2, a considerable body of evidence indicates that the transient hypoxia in the CNS after TBI is accompanied by cardiovascular sequelae, including diastolic dysfunction, increased heart rate, and elevated mean arterial pressure. These changes, which can persist for hours or even days post-injury, are hallmarks of sympathovagal imbalance (Do Nascimento et al. 2023; Leyba et al. 2023; Woodman et al. 2023).

**Table 2:** Summary of preclinical studies and main outcomes addressing the effect of TBI in the cardiac tissue

study	Species	TBI model	Purpose	Results
Leyba et al. 2023	mice swiss. Twenty-four mice (*n* = 11 females; *n* = 13 males).	Closed-head injury with long-term disorders (CHILD). Mild injury	The photoacoustic imaging (PAI) of the brain four hours and 7, 30, 90, and 240 days post-injury, as well as, 4DUS test for specific cardiac function. At the molecular level, cardiac T-p38 MAPK and troponin I (Tn1) were measured 30 days post-mTBI. A year after the injury, dobutamine stress test.	A decline in cerebrovascular sO2. Increase HbT levels 4 h following mTBI. Diastolic dysfunction and a diminished systolic strain response during stress tests, eight months following injury. Increase of T-p38 MAPK (*p* = .039) and a decrease in cardiac TnI (*p* = .005). One year following (mTBI) exhibited anxiety-like behaviors and memory impairments.
Woodman et al. 2023	C57BL/6 mice female with 2 months aged	TBI was induced by (CCI). Severe injury	Investigate the inhibition of catecholamine synthesis using alpha-methyltyrosine (αMT) one week after (CCI), as well as its effects on cardiovascular reactivity and behavioral alterations. Examine the expression of genes cortical such as the (Adra2c), the (Nkcc1), the (Kcc2) and (Ae3).	TBI increased in mean arterial blood pressure, left ventricle (LV) fractional shortening, and ejection fraction compared with sham-operated following 1 week. TBI diminished exploratory behavior during object recognition. The TBI modified the BBB marked by extravasation of fluorescein cerebral. TBI reduced the Adar2c, Kcc2 and Ae3. The Nkcc1 gene transcription was enhanced that was completely blocked by αMT.
Peng et al. 2023	adult C57BL/6 mice with 6–8 weeks aged	TBI was induced by (FPI) of pressure of 1.90 to 2 atm (moderate)	To verify whether ferroptosis is one of the important factors that causes heart damage following TBI.	TBI induce ferroptosis in cardiac tissue, evidenced by in TfR1, FPN, and GPX4 expression within a time frame of 3 h to 72 h post-TBI. Echocardiography and myocardial enzyme (serum levels of LDH, CK-MB, and CK at 24 h after TBI) revealed cardiac dysfunction after TBI. Inhibition of iron overload reduce (ROS) and p38 MAPK/NF-κB.
Qian et al. 2024	Male C57BL/6 mice with 10-week-old	TBI mouse model was induced by using a CCI	To investigate the effects of AT2R modulation and cardiac injury as well as the involvement of the immune system in cardiac injury after TBI.	Administration of C21 (AT2R agonist) improved cognitive function (MWM), (BBB). The TBI increased IL-1beta, IFN-gamma, CCL5, CCL20, CCL27, CXCL5, and CXCL10. C21 treatment improved cardiac contractility and avoid the increased of proinflammatory cytokines one month after TBI.
Wang et al. 2023	Male apolipoprotein E–deficient (ApoE−/−) on the C57BL6 with 8 weeks aged.	TBI mouse model using a CCI.	determine whether the beta-receptor antagonist, metoprolol, would be effective in preventing the increased atherosclerosis observed following (TBI) in hyperlipidemic mice.	Histological analysis revealed that TBI caused lesions in the aorta. TBI resulted in atherosclerosis. Alteration was significantly mitigated in mice that received metoprolol treatment.
Do Nascimento et al. 2023	adult male Wistar rats (350–400 g)	FPI model. Severity of below 1 ATM for mild FPI and between 1.5 and 2.0 ATM for moderate FPI	The objective of this study is to evaluate the overall heart rate variability (HRV)	Decrease in heart rate variability (HRV) activity 24 h post-(TBI). Decline of RR interval with mild and moderate injuries. The analyses demonstrated that animals with moderate impact exhibited lower RR intervals in comparison to animals with mild injury. The sympathetic-vagal activity was greater TBI.
Keane et al. 2023	C57BL/6 male mice (30 g, ~ 11 to 15 weeks-old)	CCI model	The study investigates the effects of (TBI) on cardiac health, with a particular focus on the role of the inflammasome in mediating these effects.	Increase of proteins as AIM2, ASC, and caspase-1, -8, and − 11 in the atrium of injured mice after 3 days. Elevated levels of IL-1β in the ventricles and in the cortex of mice injury.
On et al. 2021	Male Sprague-Dawley rats (8 weeks-old)	CCI model with severity moderate and severe.	Study examines the effects of traumatic brain injury (TBI) on cardiac autonomic dysfunction (CAD)	Excessive sympathetic activity in the moderate and severe TBI groups by increased excitability in stellate ganglion (SG) neurons and decreasing excitability in intracardiac ganglion (ICG) neurons. TBI reduced A-type K+ currents in neurons. M-type K+ current was increased in ICG neurons after TBI. The analysis showed that KCNQ2 was lower in SG.

**Fig. 2:**
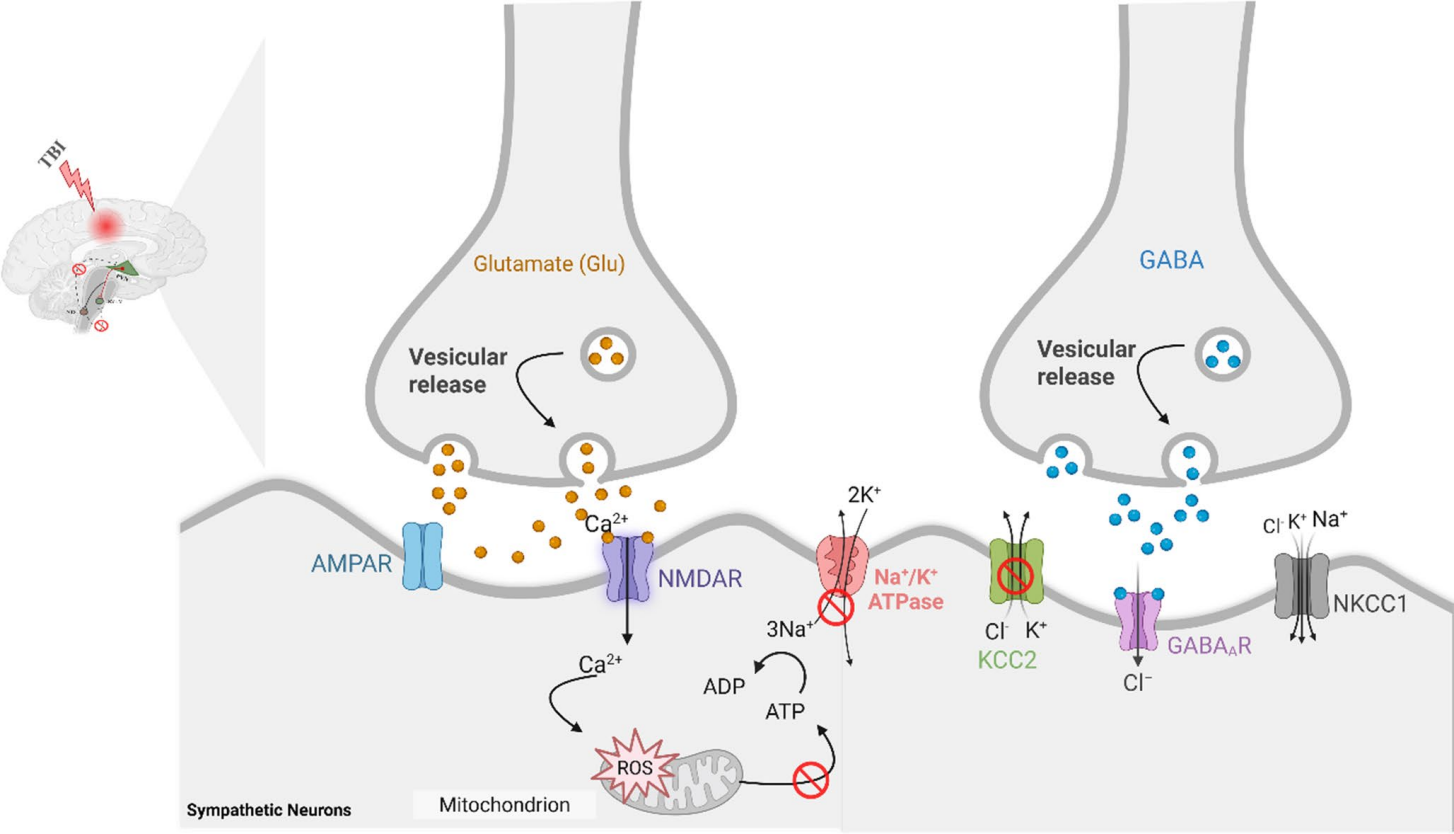
Schematic illustration of neurotoxicity effects induced by traumatic brain injury (TBI)

A key mechanism underlying TBI-induced pathophysiology is the disruption of the sympathetic nervous system. Specifically, the loss of GABAergic inhibitory tone in the paraventricular nucleus (PVN) of the hypothalamus (Fig. 2) leads to hyperactivity in the rostral ventrolateral medulla (RVLM) of the brainstem, thereby enhancing sympathetic outflow (Kim et al. 2015; Woodman et al. 2023). Under normal physiological conditions, the RVLM projects catecholaminergic fibers to preganglionic neurons in the spinal cord, which in turn regulate the atrioventricular node and myocardium. This outflow is tightly controlled by afferent signals from baroreceptors, which project to the nucleus of the solitary tract (NTS) to suppress RVLM activity and reduce sympathetic tone (Plott et al. 2024; Scalco et al. 2021).

This imbalance manifests at the cellular level as increased excitability of efferent neurons in the stellate ganglion (SG) and reduced activity in the intracardiac ganglion (ICG) (Oh et al. 2021; Wang et al. 2023). Mechanistically, this sympathetic hyperexcitability has been linked to the downregulation of KCNQ2 expression in the SG. This gene encodes a potassium channel subunit critical for K+ efflux, and its reduced expression leads to increased neuronal firing rates directed at the cardiac muscle (Oh et al. 2021). Concurrently, within the CNS, decreased expression of the alpha-2 adrenergic receptor gene (Adra2c) impairs the negative feedback control of the sympathetic system, further reinforcing the pathological interplay between the HPA axis and the heart-brain interface after TBI.

Traumatic brain injury (TBI) induces a substantial depolarization that leads to a significant release of glutamate. This release causes the hyperactivation of postsynaptic glutamate receptors, specifically N-methyl-D-aspartate (NMDA) and α-amino-3-hydroxy-5-methyl-4-isoxazolepropionate (AMPA) receptors. As a result, there is an excessive influx of intracellular Ca²⁺, which disrupts mitochondrial function and decreases the production of adenosine triphosphate (ATP). These changes reduce the activity of Na⁺/K⁺-ATPase, which is critical for maintaining the sodium and potassium ionic gradient. Under normal physiological conditions, KCC2 is expressed at higher levels compared to NKCC1, maintaining a chloride gradient across the neuronal membrane and preserving the GABAergic inhibitory tone. Following TBI, the Na⁺/K⁺-ATPase cannot maintain sodium and potassium homeostasis, leading to an up-regulation of NKCC1. Additionally, there is a reduction in KCC2 expression, which is responsible for extruding Cl⁻ from the intracellular to the extracellular space. This alteration affects the electrophysiological properties of the GABAA receptor, shifting its function from inhibitory to excitatory. This shift results in enhanced efferent sympathetic neural firing through the rostral ventrolateral medulla (RVLM) in the brainstem. Created in BioRender. Rechia Fighera, M. (2025) https://BioRender.com/81k4ghc.

**Fig. 3:**
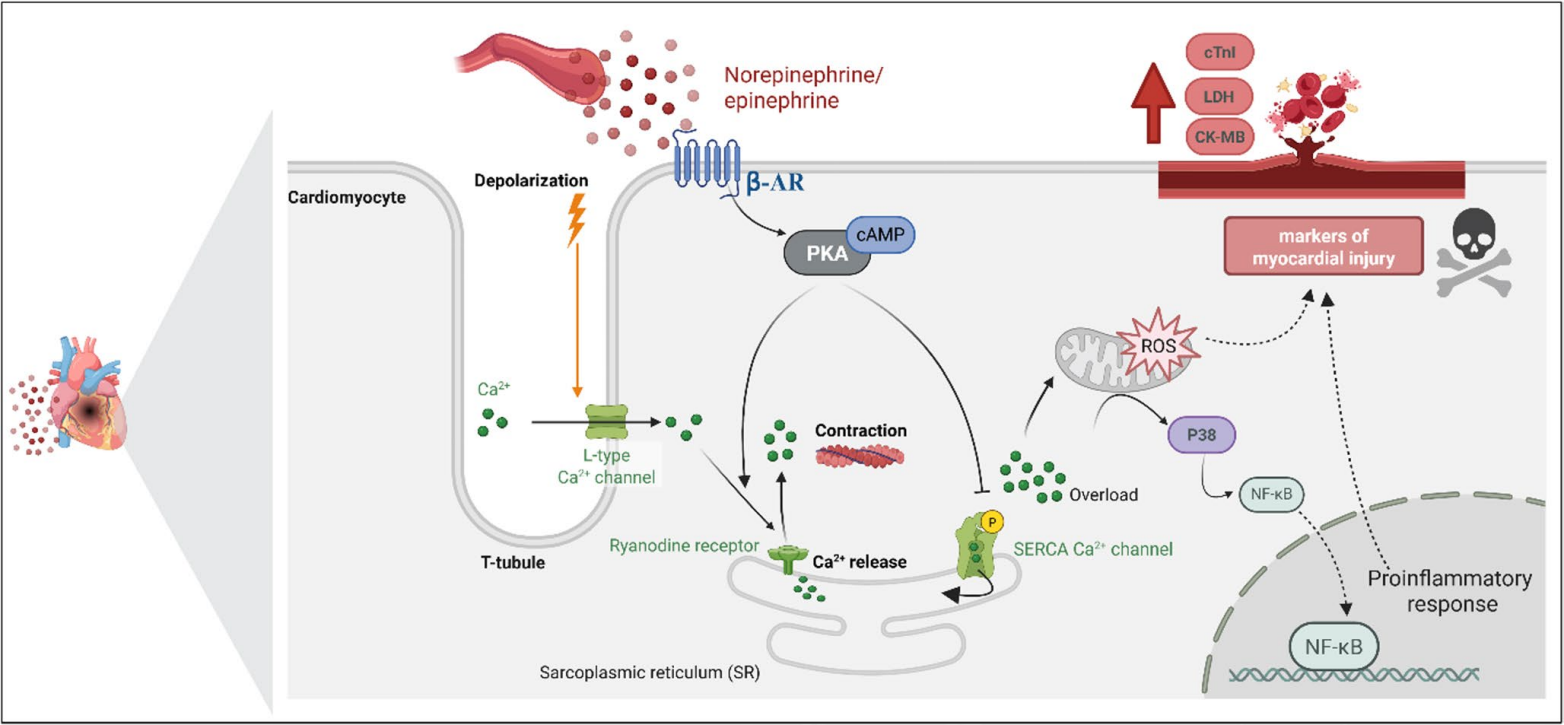
Schematic Illustration of sympathetic hyperactivity Effects Induced by TBI Following TBI, there is an enhanced efferent sympathetic neural firing that overstimulates β-adrenergic receptors, leading to the production of cyclic AMP (cAMP). This process subsequently activates protein kinase A (PKA). The depolarization of the sarcolemma facilitates cellular calcium (Ca²⁺) entry through L-type calcium channels (LTCC). The elevated cytosolic Ca²⁺ levels then trigger the release of additional Ca²⁺ from the sarcoplasmic reticulum (SR) via the ryanodine receptor 2 (RyR2) channel, further increasing intracellular Ca²⁺ concentration. Excessive catecholamines following TBI contribute to Ca²⁺ overload in the cytosol, resulting in increased oxidative stress and the activation of inflammatory pathway markers, such as p38 mitogen-activated protein kinase (MAPK) and nuclear factor kappa B (NF-κB). Consequently, there is an elevation of cardiac injury markers like lactate dehydrogenase (LDH), creatine kinase-MB (CK-MB), and cardiac troponin I (cTnI), indicating modifications in the heart-brain axis post-TBI. Created in BioRender. Rechia Fighera, M. (2025) https://BioRender.com/81k4ghc

Following TBI, this regulatory balance is lost. The resulting sympathetic hyperactivity can lead to β-adrenergic receptor desensitization, reduced cardiac contractility, and a diminished ejection fraction, ultimately increasing the risk of myocardial infarction (Li 2022). This cardiac dysfunction is reflected at the molecular level by elevated markers of myocardial injury (LDH, CK-MB, CK), increased inflammatory signaling (p38 MAPK, NF-κB), and evidence of lipid peroxidation, such as increased 4-hydroxynonenal (4-HNE) levels 24 h post-injury (Fig. 3) (Peng et al. 2023).

These pathological changes in the heart can, in turn, disrupt afferent signaling back to the brain, establishing a vicious cycle. This disruption of the heart-brain axis, where cardiovascular dysfunction exacerbates neurological deficits, likely contributes to poor long-term recovery and an increased risk of subsequent neurodegenerative and metabolic diseases (Snowden et al. 2023). The critical role of this sympathetic overactivity is underscored by pharmacological evidence. For instance, inhibiting catecholamine synthesis with α-methyltyrosine (αMT) improves both cardiac and cognitive outcomes in rodent models of TBI, while β-blocker administration has been shown to attenuate post-traumatic arteriosclerosis (Wang et al. 2023; Woodman et al. 2023).

### Reciprocal feedback mechanisms within the neuro-cardiac axis

**Fig. 4:**
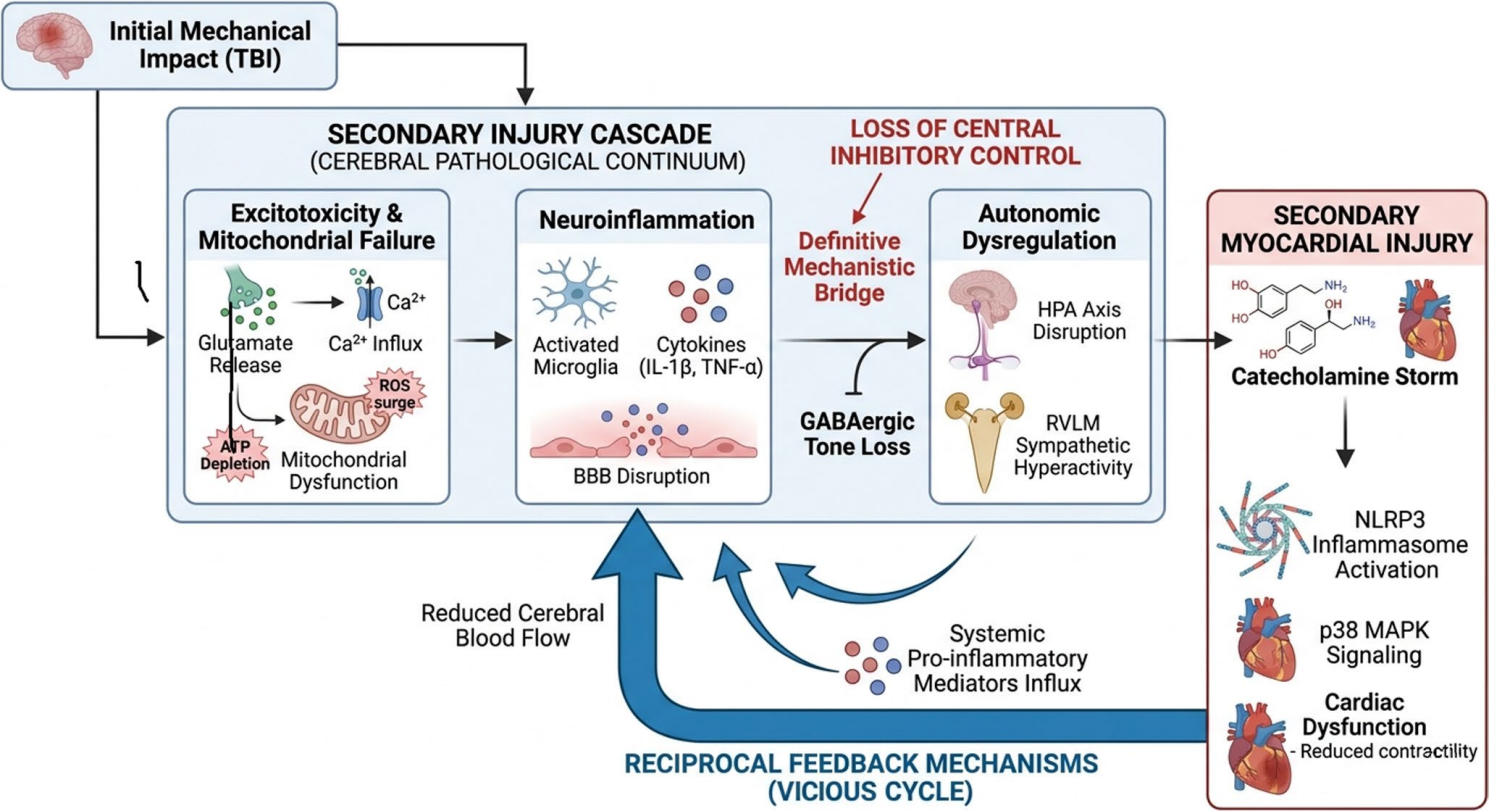
Schematic representation of the neuro-cardiac reciprocal feedback mechanisms following Traumatic Brain Injury (TBI)

After TBI, heart–brain communication is proposed to operate as a self-perpetuating vicious cycle (Fig. 4), conceptually anchored in the neuro-cardiac inflammasome axis. Within this framework, the initial neuroinflammatory response, together with the loss of GABAergic inhibitory tone in key autonomic centers (such as the PVN and NTS) and the disruption of HPA axis negative feedback, act as primary mechanistic drivers of sustained sympathetic hyperactivity. This ‘catecholaminergic storm’ causes peripheral stress and triggers systemic inflammation and oxidative damage within the myocardium via NLRP3 and p38 MAPK signaling. The resulting cardiac dysfunction and hemodynamic instability, marked by reduced cerebral blood flow and elevated pulse pressure, directly impair cerebral perfusion while exacerbating the disruption of the BBB. Such microvascular degradation facilitates a persistent influx of systemic pro-inflammatory mediators into the brain parenchyma, which consequently fuels central neuroinflammation and reinforces the underlying autonomic imbalance.

Following the initial mechanical impact, TBI triggers a secondary injury cascade that operates as an integrated cerebral pathological continuum. This cascade begins with excitotoxicity-induced mitochondrial dysfunction, triggered by excessive glutamate release, calcium influx, and increased ROS generation, ultimately leading to ATP depletion. Concurrently, oxidative stress and cellular damage establish a neuroinflammatory microenvironment, characterized by activated microglia, blood-brain barrier (BBB) disruption, and the release of pro-inflammatory cytokines such as IL-1β and TNF-α. The definitive mechanistic bridge connecting this cerebral damage to systemic dysfunction is the loss of central inhibitory control, specifically characterized by diminished GABAergic tone. This loss unleashes autonomic dysregulation, marked by HPA axis disruption and sympathetic hyperactivity in the rostral ventrolateral medulla (RVLM). Consequently, an uninterrupted catecholamine storm drives secondary myocardial injury, triggering NLRP3 inflammasome activation and p38 MAPK signaling within the cardiac tissue. This pathway culminates in cardiac dysfunction, including hypertrophy and reduced contractility. Finally, the resulting hemodynamic instability and reduced cerebral blood flow facilitate a constant influx of systemic pro-inflammatory mediators back into the brain parenchyma, thereby fueling central neuroinflammation and establishing a vicious cycle that hinders systemic recovery.

## Physical exercise in the treatment of heart diseases

### The effect of exercise on health maintenance

Sustained physical exercise remains one of the most effective strategies for preserving overall health, fostering cardiovascular endurance, muscular strength, and metabolic balance. According to current international guidelines, adults should perform 150–300 min of moderate or 75–150 min of vigorous aerobic activity weekly, together with two sessions of resistance training engaging large muscle groups (American College of Sports Medicine et al. 2022; Valenzuela et al. 2023).

Cardiorespiratory fitness (CRF) integrates cardiovascular and pulmonary efficiency, quantifying the body’s capacity for oxygen uptake and use during sustained physical effort (Prince et al. 2024). The measurement of CRF is by maximal oxygen uptake (VO₂max), normalized to body mass (e.g., mL·kg⁻¹·min⁻¹). Exercise tolerance is also described through metabolic equivalents (METs), where 1 MET corresponds to oxygen consumption at rest (3.5 mL O₂•kg⁻¹•min⁻¹). Thus, a CRF of 6 METs indicates an energy expenditure six times greater than resting levels (Fletcher et al. 2013).

Currently, about 31% of the population shows low physical activity, a figure expected to reach 35% by 2030 (Bull et al. 2020b). Epidemiological evidence reveals an inverse association between physical fitness and mortality, independent of age, sex, or race (Kokkinos et al. 2022). Consequently, sedentarism represents a major global public‑health issue linked to cardiovascular events and early death. In heart‑failure or post‑transplant patients, CRF averages < 3 METs, versus 10–13 METs in healthy adults aged 20–49. Clinically, combined assessment of CRF, blood pressure, cholesterol, and ECG can aid detection and reduction of sedentary behavior (LaMonte 2022).

#### Metabolic Adaptations of the Cardiovascular System to Physical Exercise

Physiological adaptations vary according to training modality, which is primarily classified as aerobic or resistance exercise (Tian and Meng 2019). Aerobic exercise comprises continuous, rhythmic efforts at varying intensities: light, moderate (40–59% HRR), or vigorous (60–89% HRR) (American College of Sports Medicine et al. 2022). In contrast, resistance training (RT) promotes muscle hypertrophy and strength through load-bearing, primarily via anaerobic pathways and neuromuscular adaptations (Cunha et al. 2024).

Chronic aerobic exercise promotes cardiovascular adaptations that stabilize autonomic control and metabolism. This process involves downregulation of β‑adrenergic receptors, limiting excessive sympathetic stimulation and lowering baseline heart rate. (Valenzuela et al. 2023). At the molecular level, AMPK activation stimulates insulin‑independent GLUT4 translocation and upregulates PGC‑1α, enhancing mitochondrial biogenesis, lipid oxidation, and energy efficiency (Bernardo et al. 2018; Bo et al. 2020). These adaptations vary in magnitude and specificity according to exercise intensity, which modulates metabolic stress, substrate flux, and hemodynamic load. In an experimental study using rats, (Li et al. 2024) found that 12 weeks of high‑intensity interval training (70–80% VO₂max) was associated with a greater cardiac AMPK activation than moderate‑intensity continuous training (45–55% VO₂max). Both exercise regimens similarly decreased heart rate and mean arterial pressure. Similarly, animals exposed to high-intensity interval training (HIIT, 85–90% VO₂max), but not to moderate continuous training (50–60% VO₂max), showed increased activities of antioxidant enzymes—ATP synthase, superoxide dismutase, and glutathione peroxidase—in the left ventricle after myocardial infarction (Lu et al. 2015). Collectively, these findings suggest superior cardioprotective adaptations with high‑intensity regimens, though individualized, progressive programs remain essential to ensure safety (American College of Sports Medicine et al. 2022).

Resistance training (RT) represents a potent therapeutic strategy for cardiovascular conditioning (Choi et al. 2024). The evidence indicates a ~ 17% reduction in cardiac events and favorable modulation of hemodynamic, metabolic, and lipid parameters following structured RT interventions (Paluch et al. 2024). According to meta-analytic studies, resistance training positively impacts endothelial function for both healthy individuals and patients with cardiovascular or metabolic conditions (Silva et al. 2021). This improvement appears partly mediated by suppressed endothelin‑1 (ET‑1) activity in hypertensive subjects—a peptide involved in vasoconstriction, immune regulation, and fibrotic processes (Liang et al. 2024).

In preclinical studies, ladder‑climbing protocols for 12 weeks at moderate intensity (60–75% of maximal capacity) improved cardiac contractility in post‑infarction rodents, potentially through enhanced Ca²⁺ handling in cardiomyocytes (Dias et al. 2024). Similar regimens with progressive overload (50–100% of body weight) in diabetic rodents led to improved glycemic control and attenuated cardiac inflammation, as evidenced by decreases in TNF‑α, IL‑6, Vascular Cell Adhesion Molecule‑1 (VCAM‑1), and Intercellular Adhesion Molecule‑1 (ICAM‑1), which are associated with endothelial dysfunction, atherosclerosis, and other cardiovascular complications (Yoo et al. 2024). In addition, increased endothelial nitric oxide synthase (eNOS) expression and nitric oxide (NO) contribute to these effects by enhancing vascular compliance and myocardial perfusion, thereby lowering cardiac afterload and improving oxygen delivery (Chen et al. 2022a; Roy et al. 2023; Suvorava et al. 2022). Together, these adaptations suggest that regular resistance exercise may foster endothelial resilience and mitigate risks associated with hypertension and ischemic injury.

In this regard, a meta-analytic study reported that, independent of exercise type, regular physical activity supports cardiovascular health and assists in cardiac disease rehabilitation (Taylor and Blakemore 2025). Furthermore, exercise-induced cardiac adaptations may also enhance brain health (Schuch and Vancampfort 2021). Clinical evidence shows interdependence between the heart and brain; for example, in 80 patients, heart failure and coronary artery disease were associated with brain structural alterations similar to those in Alzheimer’s disease (Mueller et al. 2020). These findings suggest that hemodynamic and neuroendocrine mechanisms triggered by exercise may preserve the structural and functional integrity of the central nervous system. However, the molecular mechanisms linking cardiac diseases to cognitive alterations, as well as the specific cardiac adaptations that benefit brain health, remain incompletely understood.

In view of this molecular gap, it is proposed that lactate produced during physical exercise induces cardiovascular and cerebral adaptations relevant to health maintenance and disease rehabilitation, acting not only as a metabolic by-product but also as a signaling molecule. In a preclinical model, lactate infusion improved hemodynamics without overloading the myocardium, via venoconstriction and arterial relaxation (Homilius et al. 2025). This effect may attenuate blood pressure and vascular stretch, which recruit glutamatergic neurons in the NTS in hypertensive contexts (Van Weperen et al. 2023), thereby reducing sympathetic tone. In parallel, in humans, post‑HIIT increases in lactate have been associated with higher cortical GABA levels (Coxon et al. 2018), possibly via increased GAD67 expression, previously reported after 4 weeks of voluntary exercise in the hippocampus, PVN, and motor cortex of rats (Hill et al. 2010). Taken together, these findings support the notion that lactate may enhance GABA levels and strengthen signaling through GABA and GABA receptors, promoting baroreceptor‑mediated bradycardia in the NTS and helping to regulate heart rate in cardiovascular dysfunction.

The exercise-induced increase in circulating lactate readily crosses the blood–brain barrier. In the central nervous system, lactate is taken up by glial cells and exported via MCT1/MCT4, after which it is transported into neurons through MCT2, where it is converted to pyruvate by lactate dehydrogenase (LDH), thereby supporting cerebral metabolic homeostasis (Homilius et al. 2025; Rae et al. 2024). In parallel, lactate enhances BDNF expression and tyrosine receptor kinase B (TrkB) phosphorylation in the hippocampus, a mechanism implicated in memory consolidation and learning, as well as in the reduction of depressive symptoms (El Hayek et al. 2019; Griego and Galván 2023). More recently, lactate has been proposed to act as a potent epigenetic modulator through a post‑translational modification (PTM) termed lactylation. This mechanism has been linked to changes in Synaptosome‑Associated Protein 91 (SNAP91), which increase the density of presynaptic vesicles, conferring resilience to chronic stress and preventing anxiety‑like behaviors (Yan et al. 2024).

We propose that exercise, by elevating circulating lactate levels, may confer cardioprotection following trauma through two complementary mechanisms: (1) direct action on the myocardium, modulating metabolism, inflammation, and oxidative stress while enhancing baroreceptor regulation via increased GABAergic tone in the brainstem; and (2) indirect action, mediated by improvements in brain function (including BDNF expression, lactylation, and neuroplasticity), which attenuate sympathetic hyperactivity and strengthen reciprocal control along the heart–brain axis.

## Physical exercise as a non-pharmacological approach to treat TBI-induced heart diseases

The pathophysiology induced by TBI presents multifaceted repercussions that vary considerably based on injury severity and individual patient characteristics. This complexity necessitates a comprehensive, interdisciplinary treatment approach to effectively minimize secondary injury cascades (Buchmann Godinho et al. 2021; Davis et al. 2024). Physical exercise emerges as a therapeutic intervention in post-TBI rehabilitation, demonstrating significant improvements across multiple domains including physical function, cardiovascular health, cognitive performance, and mental well-being (Schuch et al. 2016; Snowden et al. 2023). Epidemiological evidence demonstrates that regular physical activity correlates with reduced risk factors and decreased population mortality rates (Dovey et al. 2023). However, TBI patients face substantial physiological challenges, with studies revealing that average peak oxygen uptake typically falls below levels deemed healthy by the (ACSM) standards (Hassett et al. 2017). This decline occurs alongside numerous barriers that significantly impede recovery, including persistent fatigue, diminished motivation, cognitive impairments, and constraining environmental factors (Pham et al. 2023). In response to these challenges, rehabilitation centers that offer robust social support and foster long-term sustainability in physical activity (PA) participation are crucial components for reducing barriers and enhancing intervention effectiveness in this population (Kato et al. 2023; Quilico et al. 2022).

### The role of physical exercise in TBI patients

A significant long-term consequence of TBI is an elevated risk of chronic cardiovascular diseases, including ischemic heart disease, heart failure, and stroke (Chin et al. 2019b; Dams-O’Connor et al. 2023; Mossberg et al. 2007). This increased risk is strongly associated with poor cardiorespiratory fitness (CRF), a key indicator of the body’s ability to transport and utilize oxygen, which is often quantified by peak oxygen uptake (VO₂ peak) (Hoppeler 2018). Indeed, TBI survivors frequently present with risk factors such as hypertension, hyperlipidemia, and physical inactivity, all of which contribute to increased mortality and cognitive decline (Izzy et al. 2023). Therefore, interventions that improve CRF are critical for this population. Physical exercise is the most effective non-pharmacological strategy for enhancing VO₂ peak, and its benefits extend beyond cardiovascular health to include improvements in cognitive and mental well-being, which are also frequently compromised after TBI (Courtes et al. 2020; Schwandt et al. 2012; Todhunter-Brown et al. 2025). This positions exercise as a uniquely powerful rehabilitation strategy, capable of simultaneously addressing the cardiovascular, cognitive, and physical deficits common in TBI survivors.

Randomized trials have revealed significant cerebrovascular impairments in TBI patients, including decreased carotid arterial compliance (CAC) and increased cerebrovascular resistance (CVR) compared to healthy controls (Table 2) (Tomoto et al. 2022). The negative correlation observed between cerebral blood flow (CBF) and CVR in these patients suggests that this early vascular dysfunction contributes to the cognitive and emotional deficits common after TBI.

A key component of this pathophysiology is the disruption of the blood-brain barrier (BBB). The BBB is integral to neurovascular coupling, the process by which cerebral blood flow is precisely matched to neuronal activity, due to the close anatomical relationship between arterioles, neurons, and astrocytes. Following TBI, damage to the BBB permits the infiltration of inflammatory cells and toxic molecules into the brain parenchyma (Thorin-Trescases et al. 2018). This infiltration triggers a cascade of secondary injury, including neuroinflammation mediated by pathways such as nuclear factor kappa B (NF-κB), and oxidative stress driven by the activation of nicotinamide adenine dinucleotide phosphate (NADPH) oxidase (NOX) (Kim et al. 2024).

In contrast to the deficits observed in sedentary TBI survivors, patients who undergo aerobic exercise training (AET) show significant improvements. For instance, AET has been shown to improve VO₂ peak and reduce systemic blood pressure following TBI (Ding et al. 2021). As summarized in Table 3, the strong correlation found between VO₂ peak and neuropsychological outcomes reinforces the hypothesis that enhanced aerobic fitness can alleviate depressive symptoms in this population (Chin et al. 2015b; Schwandt et al. 2012). Furthermore, AET has been shown to improve VO₂ on-kinetics, which reflects a more rapid adjustment of oxygen uptake at the onset of exercise and is associated with reduced fatigability (Chin et al. 2019b). However, evidence regarding precise doses and responses in TBI remains scarce, thus underscoring the need for further research to establish a safe and viable intervention.

**Table 3:** Summary of clinical studies and the main outcomes of physical exercise in people with TBI

Study	Subjects	TBI severity	Intervention	Intervention details	Purpose	Results related to exercise
Ding 2021	age 18 to 65 6 months to 6 years after initial TBI	moderate to severe with GCS < 12 or amnesia > 1 day	Aerobic exercise training (AET). Stretching and toning (SAT), this group was considered as control with lower level of intensity of exercise	3 months of training. First week (20 min x 3 days) and in the end of the fourth week (30 min x 5 days or 50 min x 3 days). An increase gradually exercise intensity (from 50–60% to 70–80% of maximum HR) and this level for the remaining 8 weeks.	Cardiorespiratory fitness is measured by VO2 peak. Cognitive function	trend toward improved VO2peak in the AET group compared to the SAT group (*p* = .059). The Cohen’s d for the interaction effect on VO2peak was 1.27, indicating a large effect size. Both the groups showed a small improvement in responses cognitive function.
Chin et al. 2015	aged 33.3 ± 7.9	mild and moderate defined by the Department of Veterans Affairs/Department of Defense clinical practice guidelines	Pre-post intervention study. Aerobic exercise training program for 12 weeks. The training was realized in treadmill 3 times week.	the training was conducted in threshold of 70 to 80 heart rate with session of 30 min.	To examine the Cardiorespiratory fitness; cognitive function; depression; sleep quality	Improvement of cardiorespiratory fitness, indicated by peak Vo2 (2.2 ± 2.3mL/min/kg; *P*=.044), peak Work Rate (72 ± 47 W; *P*=.007), and Vo2 at the anaerobic threshold (2.8 ± 1.8mL/min/kg; *P*=.006). Improvement Neuropsychological assessments such as (RBANS), (TMT-A), (TMT-B) after exercise with correlations for the peak Vo2 and RBANS total scale (*r*=.831, *P*=.020),
Chin et al. 2019	Participants were included if they were between 21 and 45 years of age	non-penetrating -(TBI) of any severity at least six months prior to enrollment.	Pre-post intervention study. The experimental aerobic exercise was conducted on a standard treadmill three times per week for 30 min per session.	The exercise training was 70–80% of the heart rate (HR) of reserve with based in the baseline cardiopulmonary exercise test.	The objective of this study was to characterize and examine the VO2 kinetics of individuals with TBI before and after aerobic exercise training.	Significant improvements in oxygen uptake kinetics with reduction of fatigability, as the faster attainment of the VO2 plateau indicates a more rapid adjustment to the workload.
Schwandt et al. 2012	Individuals 18 to 55 years of age, exhibiting symptoms of depression	more than 6 months post-TBI of severity moderate to severe	Pre-post intervention study. Aerobic exercise three times per week for 12 weeks. The sessions of 30 min with predetermined power output.	The heart rate was maintained at 60% to 75% of the age-predicted maximal heart rate.	To determine the impact of an aerobic exercise program on depression symptoms among individuals with a minimum of six months of post-traumatic brain injury (TBI).	A downward trend in HAMD scores was observed among the study participants, indicating a decline in depressive symptoms. Enhancement in aerobic fitness. post intervention. Decline of heart rate in the same power. The Rosenberg Self-Esteem Scale revealed an enhancement in their overall self-esteem post intervention
Tomoto et al. 2022	men and women aged between 18 and 65 with closed mechanism of injury; 6 months to 6 years after the incident TBI	TBI of moderate to severe severity Glasgow Coma Scale less than 12, and neuroradiological findings of injury	The TBI group was randomly assigned to either the AET group or the SAT group for a 3-month period.	First week − 3 exercise sessions for 20 min each at 50% to 60% of the maximal HR. In weeks 2 to 4, the exercise intensity of 70% to 80% of maximal heart rate, with (30 min × 5 days or 50 min × 3 days) until end study	measure Carotid arterial compliance (CAC) at baseline and after AET, as well as, cerebral blood flow (CBF); cerebrovascular resistance (CVR)	Individuals with TBI had lower CAC (*P* = .046). Exhibited higher (CVR) compared to controls. After 3 months of AET, there was a tendency for CAC to increase compared to a SAT group (*P* = .080) and improved of VO2 max (*P* = 0,062).

### Exploring the molecular adaptations of physical exercise in cardiac tissue after TBI

By inducing physiological and molecular adaptations that support health, physical exercise offers non‑pharmacological therapy for cardiovascular and psychiatric conditions and a viable rehabilitation avenue for TBI survivors with co‑occurring cardiac and behavioral impairments (Chen et al. 2022b; Schuch et al. 2016). Its cardioprotective actions after TBI are thought to depend largely on exercise‑induced tuning of autonomic circuits and neuro‑cardiac communication, although the exact molecular pathways remain to be clarified.

One potential mechanism involves the modulation of inflammatory pathways. For example, in a model of cardiac dysfunction induced by the β-adrenergic agonist isoproterenol, exercise conferred protection via the IL-10/STAT3 signaling cascade. In this pathway, exercise stimulates macrophages to secrete the anti-inflammatory cytokine Interleukin-10 (IL-10), as summarized in Table 4. IL-10 then activates the STAT3 transcription factor, which promotes the expansion of myeloid-derived suppressor cells (MDSCs). These MDSCs, in turn, contribute to improved cardiac function and reduced pathological remodeling (Feng et al. 2022; Yang et al. 2020).

**Table 4:** Summary of preclinical studies and main outcomes addressing the effect of physical exercise in the cardiac tissue

Study	Species	Exercise protocols	Purpose	Results
Yang et al. 2020	sprague dawley male rats	First week of acclimatization in the treadmill. Exercise training of with treadmill speed of 9 m/min, 40 min per day in the week 1. After, treadmill speed 12 m/min per 60 min day for 4 week	Ascertain whether the morphologic and echocardiographic alterations were ameliorated by exercise treatment. To determine the concentration of enzymes of cardiac injury.	Exercise reduced the cytokines ( IκBα, NF-κB, COX-2, IL-8, TGF-β1, phosphorylated ERK, Sp1) doxorubicin-induced. Exercise reduced the expression of several cardiac remodeling-associated factors, including FGF2, uPA, MMP-2, and MMP-9. The LDH and CK-MB levels also were reduced.
Feng et al. 2022	Male C57BL/6 mice (6–8 weeks old)	Initially, each session lasted 1.5 h, with an incremental increase of 15 min each day, culminating in a duration of 2.5 h on the fifth day.	To investigate the protective effects of exercise training (ET) on heart failure (HF).	The ET enhanced cardiac function in mice, Myeloid-Derived Suppressor Cells (MDSCs) with isoproterenol-induced heart failure. ET activated the secretion of (IL-10) from macrophages. This activation was through STAT3/S100A9 signaling pathway, achieving the heart protection.
Raquel et al. 2023	Male Wistar rats (8 weeks old)	rats were adapted to walk/run on a treadmill for 5 day. Training intensity of (50–60% of maximal intensity, 1 h/day, 5 days/week) for 8 weeks.	To investigate the impact of blood-brain barrier (BBB) lesions on autonomic imbalance in heart failure (HF) and the effects of exercise training.	The HF rats exhibited increased BBB permeability in autonomic areas by expression of caveolin-1 and reduced tight junction (TJ) Conversely, exercise training was shown to displace caveolin-1 and increase claudin-5, thus reducing BBB permeability.
Bartra et al. 2022	Young adult male mice C57BL/6J	Sedentary (SED), Moderate physical activity (MOD): 15 cm/s and 5 degrees positive slope for 30 min/day. Intense physical activity (INT): 30 cm/s and 10 degrees of positive slope for 45 min/day. Training was carried out 5 days/weeks, for 8 weeks.	The effects of exercise on cardiovascular and cognitive functions in young adult male mice	high-intensity training resulted in a decrease in heart rate. There was significant decrease after intensity exercise of proteasome subunit beta 2. Nfe2l2 and Sod2 expression was maintained, but Cat expression was significantly enhanced in INT mice.
Tan et al. 2023	Adult male C57BL/6 mice	Mice in the exercise group were adapted to swimming training. Mice with established MI (4weeks post-surgery) were subjected to different doses of swimming exercises (0, 15, 30, and 60 min/day for 8 weeks)	the effects of different doses of exercise on cardiac function in male mice with established myocardial infarction (MI)	Exercise (15 min per day) improved cardiac function and reduced mortality in mice with established MI. Exercise of 15 min upregulate the expression of (CTRP9) and circulating insulin-like growth factor 1 (IGF-1) in the heart. Moderate and high doses of exercise did not confer a protective effect against established MI.

Beyond direct neuronal modulation, exercise influences the renin-angiotensin-aldosterone system (RAAS). The primary effector of this system, angiotensin II, acts through two opposing receptors: The angiotensin type 1 receptor (AT1-R) and the Angiotensin Type 2 Receptor (AT2-R). Broadly, AT1-R activation is pathogenic, promoting vasoconstriction, hypertension, inflammation, and oxidative stress. In contrast, AT2-R activation is protective, mediating vasodilation and anti-inflammatory effects (Dasgupta and Zhang 2011; Ksiazek et al. 2024). Crucially, AT2-R helps counterbalance the sympathetic drive driven by AT1-R, as it is highly expressed on GABAergic neurons within the NTS, directly linking the RAAS to central inhibitory control (Elsaafien et al. 2020). Supporting these neurocardiac protective role of AT2‑R (Qian et al. 2024) reported that C21, an AT2‑R agonist, preserved blood–brain barrier (BBB) integrity and attenuated cerebral and cardiac pro‑inflammatory protein expression in animals subjected to TBI. Consistent with the therapeutic potential of RAAS modulation, (Xue et al. 2022) reported that 12 weeks of voluntary exercise in rats reduced pro‑hypertensive mediators, including AT1‑R, ACE1, IL‑1β, and TNF‑α, in the hypothalamic PVN. In parallel, the same study observed increased AT2‑R mRNA and IL‑10 expression, highlighting the cardioprotective impact of regular exercise. Based on these considerations, we propose that physical exercise may serve as a potent non‑pharmacological approach to RAAS modulation in brain tissue and myocardium following TBI. This effect would likely occur via decreased angiotensin II production and reduced AT1-R activation, accompanied by upregulation of AT2-R. However, additional experimental and clinical investigations are needed to confirm this proposition.

As previously noted, it is proposed that physical exercise may help restore the bidirectional communication of the heart–brain axis after TBI. Supporting this assumption, a study in rats with cardiac dysfunction reported that treadmill training at 50–60% intensity for 8 weeks reduced BBB permeability in brain regions involved in autonomic regulation, such as the PVN, NTS, and RVLM (Raquel et al. 2023). In addition, a cycling protocol in rats (60 min daily for 28 days) contributed to the restoration of chloride homeostasis after spinal cord injury by upregulating the KCC2 transporter and downregulating NKCC (Cote et al. 2014). These adaptations facilitate Cl⁻ efflux, thereby enhancing GABA receptor activation and the tone of the GABAergic system. Taken together, these effects may be beneficial in counteracting excessive glutamate release after trauma, as well as in re‑establishing the balance between sympathetic and parasympathetic outflow in the context of cardiac dysfunction.

### Exploring lactylation as a molecular mediator in the heart-brain axis

**Fig. 5:**
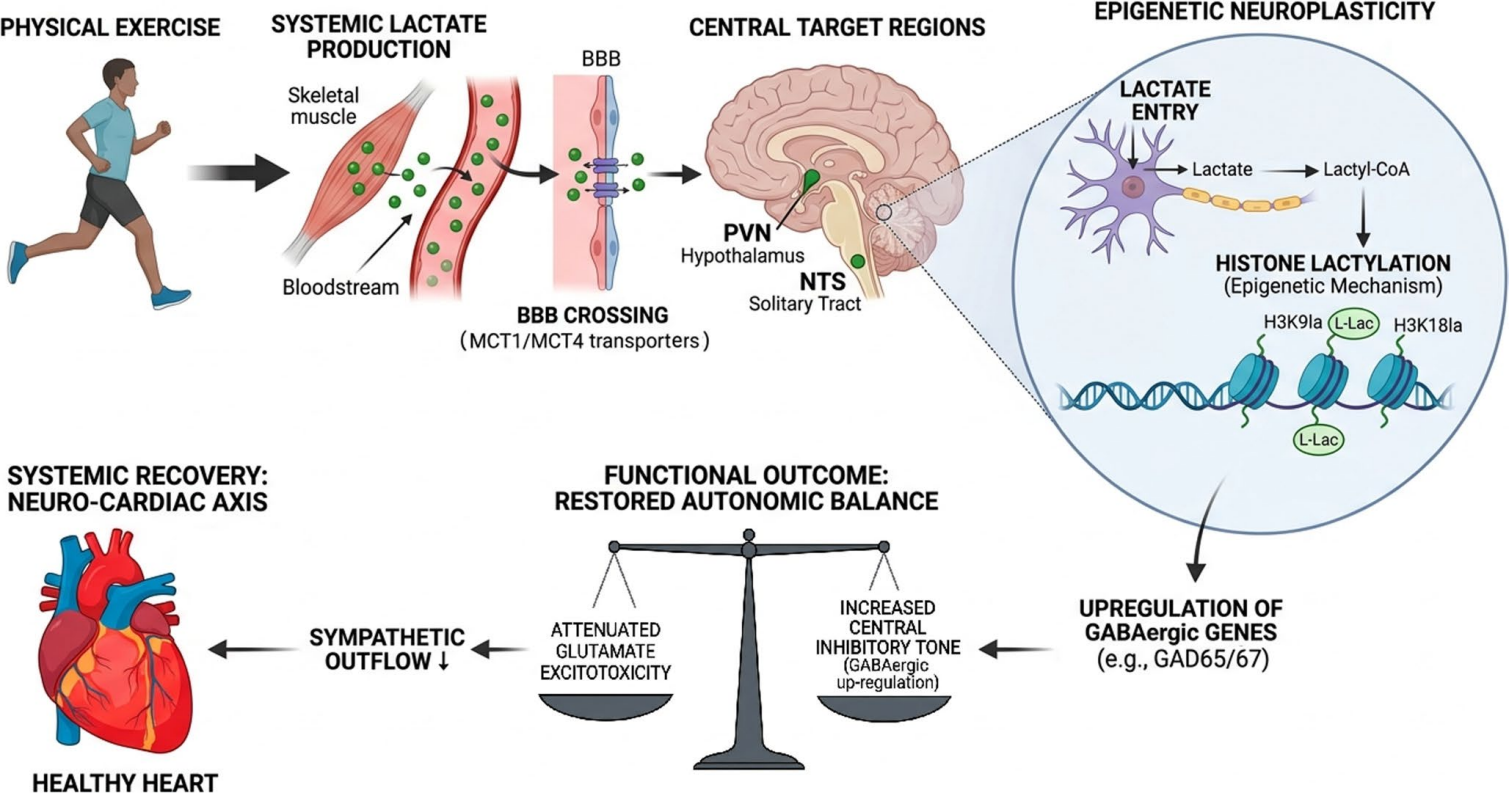
Proposed mechanistic framework of exercise-induced epigenetic neuroplasticity and the restoration of the neuro-cardiac axis

To address current gaps in the understanding of heart–brain communication following TBI, a novel conceptual framework is proposed, centered on exercise-induced epigenetic modifications (Fig. 5). Within this framework, physical exercise induces a marked systemic increase in circulating lactate, which readily crosses the blood–brain barrier and reaches key autonomic control centers, including the paraventricular nucleus (PVN) and the nucleus of the solitary tract (NTS), thereby linking peripheral metabolic signals to central regulatory mechanisms.

Within these microenvironments, lactate acts as a metabolic substrate and an epigenetic modulator through histone lactylation, a mechanism that directly regulates gene transcription (Zhang et al. 2019). This exercise-induced lactylation may specifically upregulate the expression of components within the GABAergic system, thereby increasing central inhibitory tone. This mechanism is critical because enhanced GABAergic signaling directly counterbalances the persistent excitotoxicity and excessive glutamate release triggered by the initial mechanical trauma. Furthermore, recent evidence highlights that brain lactylation pathways directly modulate neuro-cardiovascular circuits and inflammatory responses, being a key driver in conditions such as neurogenic hypertension (Wei et al. 2024).

Consequently, the epigenetic restoration of inhibitory tone dampens hyperactive sympathetic outflow and reactivates parasympathetic control. By explicitly positioning lactylation as a key molecular mediator, this framework offers a distinctly novel perspective: it delineates how peripheral metabolic signals generated during exercise translate into central neuroplastic changes, ultimately rescuing the bidirectional communication within the neuro-cardiac axis.

In this hypothesized model, physical exercise promotes a significant systemic increase in circulating lactate produced by skeletal muscle. We propose that this circulating lactate efficiently crosses the blood-brain barrier (BBB) and reaches critical autonomic control centers, specifically the paraventricular nucleus (PVN) of the hypothalamus and the nucleus of the solitary tract (NTS). Within these microenvironments, neuronal uptake of lactate is suggested to act not merely as a metabolic substrate, but as a epigenetic modulator through histone lactylation. We hypothesize that this specific exercise-induced epigenetic modification may upregulate the expression of GABAergic genes, thereby increasing central inhibitory tone to directly counterbalance TBI-induced glutamate excitotoxicity. Consequently, the epigenetic restoration of central GABAergic tone would dampen hyperactive sympathetic outflow and restore overall autonomic balance. Ultimately, we propose that this molecular mechanism could mitigate secondary myocardial injury and rescue the bidirectional communication within the neuro-cardiac axis, facilitating systemic cardiovascular recovery.

#### Considerations for exercise prescription in the TBI population

Prescription of exercise for individuals with TBI should be carefully individualized, given the multifactorial nature of this condition. Severity, symptom fluctuation, environmental constraints, personal characteristics, and motivation decisively modulate the training response (Vuu et al. 2025). It is important to emphasize that, in the acute and subacute phases, safe progression of exercise intensity remains widely debated, as high-intensity or poorly dosed training may exacerbate symptoms and hinder rehabilitation (Weldon et al. 2024b).

Although the therapeutic potential of physical exercise is increasingly supported by clinical evidence, translating these neurobiological benefits into clinical practice requires rigorous precision to ensure patient safety. Before initiating any progressive training, clinicians must systematically evaluate the patient for absolute and relative cardiovascular red flags. Based on the American College of Sports Medicine (ACSM) guidelines and the Buffalo Concussion Treadmill Test (BCTT) safety protocols, exercise testing and prescription are strictly contraindicated in the presence of acute myocardial infarction (within the preceding two days), high-risk unstable angina, symptomatic severe aortic stenosis, acute pulmonary embolus, and uncontrolled symptomatic heart failure (American College of Sports Medicine et al. 2022; Leddy 2025).

Relative contraindications that warrant specialized medical evaluation prior to mobilization include severe arterial hypertension, defined as resting systolic blood pressure exceeding 200 mmHg or diastolic blood pressure exceeding 110 mmHg, and uncorrected electrolyte imbalances (Leddy 2025).In the acute and subacute phases of moderate-to-severe TBI, patients are frequently managed in neurointensive care or specialized inpatient rehabilitation. In these settings, clinical precision dictates that active physical therapy must only proceed when the patient has achieved objectively defined markers of hemodynamic stability. For instance, active mobilization is contraindicated, and should be immediately interrupted, if the Intracranial Pressure (ICP) exceeds 22 mmHg for more than 5 min without external stimulation, or if the Cerebral Perfusion Pressure (CPP) cannot be consistently maintained above the critical threshold of 60 mmHg (Jackson 2023).

These quantitative boundaries align with the foundational principles of the 2025 BRIDGES (Brain Injury: developing guidelines for physical activities) clinical practice guidelines, which emphasize the absolute necessity of rigorous physiological monitoring and individualized pre-exercise screening for the moderate-to-severe TBI population (Hassett et al. 2025). For individuals recovering from mild TBI and concussion, safety hinges entirely on precise, real-time symptom monitoring. Utilizing validated protocols such as the BCTT, clinical precision requires non-negotiable stopping criteria to prevent the exacerbation of dysautonomia. Prior to initiating an exercise session, if a patient reports a baseline overall symptom severity of 7 or higher on a 10-point scale, exercise is temporarily contraindicated (Leddy 2025). During the exertion phase, the session must be immediately terminated if the patient experiences an acute symptom exacerbation representing an increase of 3 or more points compared to their resting baseline (Leddy 2025). Additionally, the test or therapy must cease if the patient reaches their age-predicted maximal heart rate or exhibits a Borg Rating of Perceived Exertion (RPE) exceeding 18, indicating maximal exhaustion (Leddy 2025).

Once these rigorous safety parameters and clinical stability criteria are met, the literature supports that early initiation of submaximal aerobic exercise (2–14 days post-concussion) may significantly reduce the occurrence of persistent post-concussion symptoms in mild TBI (Leddy et al. 2023). In contrast, for moderate-to-severe TBI, while there is no universally defined timeframe for initiating interventions, achieving the aforementioned hemodynamic and intracranial stability is the absolute prerequisite for mobilization (Hassett et al. 2025).

Therefore, it is essential to clearly distinguish the specific clinical considerations for prescribing exercise in concussion or mild TBI (Box 1) from those applied to moderate-to-severe TBI (Box 2). Accordingly, the subsequent sections summarize core recommendations from international consensus statements and guidelines into boxes, offering a practical and safe overview of the principal prescription parameters currently available.

**Table:** Box 1: Considerations for exercise prescription following mild traumatic brain injury or concussion

1- After 2 days of relative rest, initiate sub‑symptom threshold aerobic exercise guided by heart rate.
2- Intensity should be progressed in two stages: light (approximately 55% of maximal heart rate) and moderate (approximately 70% of maximal heart rate). Progression to the moderate stage is recommended after 24 h of successful light exercise (i.e., without symptom exacerbation).
3- Use the 0–10 Visual Analogue Scale (VAS) to monitor symptom exacerbation during exercise (active management). Increases in symptoms should not exceed 2 points above the value recorded at rest.
4-If, during rehabilitation, the patient reports symptoms exceeding 2 points above baseline on the VAS, the session should be discontinued, and training should be resumed the following day at an intensity below the workload at which symptoms occurred.
5- Any activity that increases the risk of head impact should be avoided until medical clearance has been obtained and symptoms have fully resolved.
The best‑practice recommendations outlined in this review are consistent with the guideline “Consensus statement on concussion in sport: the 6th International Conference on Concussion in Sport – Amsterdam, October 2022”, (Patricios et al. 2023) and “Sport-Related Concussion”, (Leddy 2025). Complementary reading of these documents is recommended for clinicians and researchers who intend to implement these guidelines in clinical practice and/or research settings.

**Table:** Box 2: Considerations for exercise prescription following moderate-to-severe traumatic brain injury

1- The implementation of individualized ballistic resistance training is strongly advocated to enhance functional mobility and muscular strength in adult and older populations.
2- Mobility training tailored to specific tasks, grounded in motor learning principles, is advised, as direct injuries to neuromotor units and prolonged periods of rest can impair mobility. These recommendations apply to adults and older adults.
3- For cardiorespiratory fitness, pre-exercise screening and physiological monitoring—such as heart rate and blood pressure assessment—are essential to ensure safety and facilitate individualized prescriptions.
4- Aerobic exercise should be performed at least three times per week for 20 to 40 min at an intensity of 40–80% of heart rate reserve. A Borg scale rating not exceeding 13/20 is suggested.
5- For children and adolescents, both strength and mobility training should incorporate playful elements and games to promote physical activity more effectively.
The best‑practice recommendations outlined in this review are consistent with the guideline “A Physical Activity Clinical Practice Guideline for People With Moderate to Severe Traumatic Brain Injury”, (Hassett et al. 2025) and “ACSM’s guidelines for exercise testing and prescription”, (American College of Sports Medicine et al. 2022). Clinicians and researchers wishing to implement these guidelines in clinical practice and/or research contexts are encouraged to undertake a complementary reading of these documents.

It is noteworthy that the symptoms outlined in Box 1 encompass headache, dizziness, nausea or vomiting, visual disturbances (such as blurred or double vision and photophobia), phonophobia (increased sensitivity to sound), fatigue, and affective or emotional manifestations. In summary, despite these advances, there remains a clear need for robust, high‑quality randomized clinical trials. Such trials are necessary to further refine exercise rehabilitation protocols for individuals with post‑concussion symptoms. In particular, they should help delineate safe and effective intensity thresholds. These thresholds need to be informed by objective physiological markers, such as blood lactate and VO₂max, especially in the context of early rehabilitation through physical exercise.

Patients with moderate‑to‑severe traumatic brain injury (TBI) commonly undergo lengthy hospital admissions, frequently requiring intensive care for periods ranging from days to months. Such trajectories are usually accompanied by sustained bed rest, leading to deterioration in motor coordination, reductions in muscle mass, and impaired cardiorespiratory fitness (Hassett 2023). Therefore, physical training should be initiated as early as clinical stability permits, consistent with the guidance summarized in Box 2.

In addition to the considerations outlined in Box 2, the duration of exercise-based rehabilitation treatment warrants careful attention to achieve consistent therapeutic effects. Recent evidence suggests that 20 weeks of exercise intervention in patients with severe traumatic brain injury (TBI) yields significant improvements in cognitive testing, whereas shorter protocols of 4 to 12 weeks fail to produce comparable impacts (Bateman et al. 2001; Lee et al. 2014; López et al. 2024). Ultimately, future research should prioritize the development of protocols that define the optimal duration for aerobic exercise to yield significant effects in individuals with moderate to severe traumatic brain injury.

## Conclusion

In conclusion, this review has underscored the critical and bidirectional nature of the heart-brain axis in the pathophysiology of traumatic brain injury. The evidence presented demonstrates that TBI is not merely a neurological event but a systemic condition, where secondary injuries to peripheral organs, particularly the heart, significantly hinder patient recovery. We have identified autonomic nervous system dysfunction, driven by profound sympathetic hyperactivity, as the primary mediator of these adverse cardiovascular outcomes, which include increased heart rate, elevated peripheral vascular resistance, and compromised cerebral blood flow.

Mechanistically, this review posits that the loss of central inhibitory control is fundamental to this process. We propose that a reduction in GABAergic signaling within key autonomic centers (e.g., the hypothalamus, RVLM, and NTS) unleashes the sympathetic hyperactivity that drives cardiac pathology. Compounding this, HPA axis dysregulation contributes to a state of systemic inflammation, fueled by increased blood-brain barrier break and intestinal permeability, which further exacerbates cardiac damage through elevated oxidative stress.

In this context, physical exercise emerges as a uniquely promising non-pharmacological intervention. By targeting the very mechanisms discussed, restoring autonomic balance, enhancing central inhibitory tone, and rebalancing neuro-hormonal systems, exercise has the potential to simultaneously address the cardiovascular, cognitive, and physical deficits common in TBI survivors. Ultimately, we outline key considerations drawn from international guidelines and consensus statements to deliver practical orientation for healthcare professionals and researchers. Thereby, a deeper understanding of these exercise-induced adaptations is essential for translating physiological insights into targeted rehabilitation strategies that can improve long-term outcomes and quality of life for this vulnerable population.

## Future directions

This narrative review, while synthesizing crucial evidence, unequivocally underscores the urgent need for rigorous future research to unlock the full potential of exercise in post-TBI recovery and clinical application. Advancing this critical field demands our focused attention on several key future directions:

### Elucidating Definitive Molecular Mechanisms

Future experimental studies must definitively confirm the intricate involvement of epigenetic alterations, particularly lactylation processes, in modulating specific brain centers that regulate the cardiac system following TBI. This mechanistic clarity is paramount for targeted therapeutic development.

Identifying Robust Translational Biomarkers: A critical need exists to identify and validate cardiac and cognitive biomarkers that are most sensitive and reliable for assessing the efficacy of diverse exercise protocols. This includes exploring lactate profiles, GABAergic system activity, and HPA axis dynamics as potential indicators for modulating both brain function and the cardiovascular system after traumatic brain injury. Such biomarkers are essential for bridging the translational gap.

Optimizing Aerobic Exercise Dose-Response for Heterogeneous TBI: A fundamental challenge lies in determining the optimal duration and intensity of aerobic exercise required to maximize the beneficial effects of lactate in individuals with moderate to severe TBI. Future research must meticulously consider the inherent heterogeneity of the injury, individual patient responses, and potential genetic or environmental modifiers to establish personalized, evidence-based exercise prescriptions.

## Limitations

Despite the valuable insights generated, several translational challenges concerning the extrapolation of long-term autonomic outcomes from preclinical data to human pathology must be acknowledged. A primary consideration is the difference in biological timescales between models and human patients. For instance, Paroxysmal Sympathetic Hyperactivity (PSH) is a syndrome of simultaneous, paroxysmal transient increases in sympathetic and motor activity that can persist for weeks or months in humans after severe acquired brain injury (Meyfroidt et al. 2017). Conversely, preclinical research mapping the windows of cognitive deficits, such as spatial learning and working memory impairments, shows these deficits frequently resolve or reach a performance plateau around 15 to 60 days post-injury in rodents (Paterno et al. 2017). Therefore, caution is required when directly equating an autonomic recovery or performance stabilization observed within a short post-injury window in a murine subject to long-term neuro-cardiac homeostasis in humans.

Furthermore, the translation of preclinical outcomes must consider the differences in care environments, as standard small animal models do not typically incorporate advanced neurocritical care (Neuro-ICU) interventions. To address this translational gap, large animal models such as swine are now being integrated with clinical critical care pathways (O’Donnell et al. 2023). Clinical survivors of moderate-to-severe TBI are routinely subjected to interventions aimed at managing cerebral perfusion pressure, including sedation, mechanical ventilation, and the use of hyperosmolar therapies like hypertonic saline (O’Donnell et al. 2023). These clinical maneuvers actively influence systemic physiological tone, whereas the outcome documented in a standard rodent model typically reflects the unmitigated natural history of the secondary injury cascade.

Ultimately, while indispensable for evaluating acute cellular and molecular mechanisms, existing small preclinical models present anatomical, temporal, and environmental boundaries when predicting human long-term systemic responses to interventions. Future research prioritizing large-animal gyrencephalic models integrated with critical care parameters will be highly valuable to continue bridging this translational gap (O’Donnell et al. 2023). 

## Data Availability

No datasets were generated or analysed during the current study.
